# Physical Activity Is not Associated with Estimated Glomerular Filtration Rate among Young and Middle-Aged Adults: Results from the Population-Based Longitudinal Doetinchem Study

**DOI:** 10.1371/journal.pone.0133864

**Published:** 2015-10-14

**Authors:** Gerrie-Cor M. Herber-Gast, Gerben Hulsegge, Linda Hartman, W. M. Monique Verschuren, Coen D. A. Stehouwer, Ron T. Gansevoort, Stephan J. L. Bakker, Annemieke M. W. Spijkerman

**Affiliations:** 1 Centre for Nutrition, Prevention and Health Services, National Institute of Public Health and the Environment, Bilthoven, The Netherlands; 2 Department of Internal Medicine and Cardiovascular Research Institute, Maastricht University Medical Center, Maastricht, The Netherlands; 3 Julius Center for Health Sciences and Primary Care, University Medical Center Utrecht, Utrecht, The Netherlands; 4 Department of Nephrology, University Medical Center Groningen, University of Groningen, Groningen, The Netherlands; The University of Tokyo, JAPAN

## Abstract

There is debate as to whether physical inactivity is associated with reduced kidney function. We studied the prospective association of (changes in) physical activity with estimated glomerular filtration rate (eGFR) in adult men and women. We included 3,935 participants aged 26 to 65 years from the Doetinchem Cohort study, examined every 5 years for 15 years. Physical activity was assessed at each round using the Cambridge Physical Activity Index. Using the CKD-EPI (Chronic Kidney Disease Epidemiology Collaboration) equation, GFR was estimated from routinely measured cystatin C concentrations, examining all available samples per participant in one assay run. We determined the association between 1) physical activity and eGFR and 2) 5-year changes in physical activity (becoming inactive, staying inactive, staying active, becoming active) and eGFR, using time-lagged generalized estimating equation analyses. At baseline, 3.6% of the participants were inactive, 18.5% moderately inactive, 26.0% moderately active, and 51.9% active. The mean (± SD) eGFR was 107.9 (± 14.5) mL/min per 1.73 m^2^. Neither physical activity nor 5-year changes in physical activity were associated with eGFR at the subsequent round. The multivariate adjusted βeGFR was 0.57 mL/min per 1.73 m^2^ (95% Confidence Interval (CI) -1.70, 0.56) for inactive compared to active participants. Studying changes in physical activity between rounds, the adjusted βeGFR was -1.10 mL/min per 1.73 m^2^ (95% CI -4.50, 2.30) for those who stayed inactive compared with participants who became active. Physical activity was not associated with eGFR in this population-based study of adults.

## Introduction

Chronic kidney disease (CKD) is recognised as a major global public health problem [1, 2]. The prevalence of CKD has been estimated to be as high as 10–16% in the general population [3–6]. In order to reduce the burden of CKD, it is essential to identify modifiable lifestyle factors that, when intervened upon, may help to prevent disease progression. Physical activity has been suggested to be one of those factors. Lack of physical activity has been linked to reduced estimated glomerular filtration rate (eGFR) and a higher prevalence of end-stage kidney disease [7–9] in cross-sectional analyses of population-based cohorts. Few studies have however investigated the prospective associations between physical activity and kidney function, and their findings are contradictory. In a study of older adults, higher levels of physical activity were associated with a lower risk of rapid kidney function decline [10]. In the NHANES II study, physical inactivity was associated with increased risk for end-stage kidney disease and CKD-related death [11]. Physical activity was however not associated with 5-year incidence of a low eGFR in a study of adults 25 years or older [12]. However, in all of these studies kidney function was already impaired at baseline and limited data are available on physical activity in people with well-preserved kidney function. Furthermore, previous studies estimated kidney function with serum creatinine, which is positively associated with muscle mass. Serum creatinine levels may therefore be falsely increased in physically active individuals with higher muscle mass. Therefore, studies using cystatin C, which is independent of muscle mass [13], as a measure of kidney function are warranted. Additionally, although physical activity is likely to change over time [14, 15], the previous prospective studies used a single measure of physical activity only. The effects of changes in physical activity on kidney function therefore remain unknown. Studying this may provide valuable information about high-risk groups and potential interventions. We investigated the longitudinal associations of physical activity and changes in physical activity with kidney function in a general population sample of men and women.

## Subjects and Methods

### Study setting

The Doetinchem Cohort Study is a longitudinal population-based study of men and women from the town of Doetinchem, located in a rural area in the eastern part of the Netherlands. In total, 12,405 participants (response rate 62%), aged 20–59 years at baseline, were first examined, using self-completed questionnaires and a physical examination, in 1987–1991 (R1). Of those, a random sample of 7768 participants was reinvited to be examined in 1993–1997 (R2), 1998–2002 (R3), 2003–2007 (R4) and 2008–2012 (R5). The response rate for the second, third, fourth and fifth round was ≥ 75%. Full details of the recruitment and response rates are reported elsewhere [16]. All participants gave written informed consent. The external Medical Ethics Committee of the Netherlands Organization of Applied Scientific Research (TNO) approved the study.

### Study population

Since physical activity data were not collected at R1 and in 1993 at R2, we included participants from 1994 in R2 onwards (n = 5016). In this study, 1) physical activity and 2) 5-year changes in physical activity were used as the main exposures. For the physical activity analysis, we excluded those who did not respond to at least two sequential rounds (n = 1038) or did not have complete data on physical activity, covariates and kidney function for at least two rounds (n = 43), leaving 3935 participants for this analysis. Similarly, after exclusion of participants who did not respond to at least three sequential rounds (n = 1757) or did not have complete data on exposures and outcome for at least three rounds (n = 39), 3220 participants were included in the additional analysis of changes in physical activity analysis versus kidney function. Pregnant women were censored at the round when they reported to be pregnant.

### Kidney function

In all rounds, a 30-ml nonfasting plasma blood sample was drawn. Creatinine was measured by dry chemistry (Eastman Kodak, Rochester, NY). Furthermore, cystatin C was based on a particle enhanced-turbidimetric immunoassay using reagents from Gentian (Gentian, Moss, Norway). Per participant, all available samples from consecutive rounds were examined in one assay run, thereby reducing the chance of measurement error to an absolute minimum[17]. eGFR was estimated with the CKD-EPI (Chronic Kidney Disease Epidemiology Collaboration) equation [18] using cystatin C for the main analysis and creatinine for a sensitivity analysis. Physical activity may influence serum creatinine levels via changes in muscle mass. Unlike the previous studies, we therefore used cystatin C to estimate kidney function for our primary analysis, which is less dependent on muscle mass.

### Physical activity

In all rounds, physical activity was self-reported using the validated [19] European Prospective Investigation into Cancer and Nutrition (EPIC) questionnaire. The questions refer to activity in the past year. Physical activity at work was first assessed and categorized as: sedentary (including currently unemployed participants), standing (e.g., hairdresser, shop assistant, and guard), physical job (e.g., plumber, cleaner, and nurse), or heavy manual job (e.g., dock worker, construction worker, and bricklayer). The respondents were then asked about the amount of time (in h/wk) during winter and summer spent in each of the following activities: walking, cycling, gardening, do-it-yourself activities, physical exercise and housework. The average time spent in recreational activity per day was estimated as the mean of the self-reported total hours per week during winter and summer divided by seven. To create the Cambridge Physical Activity Index (CPAI), data on physical activity at work and leisure time were combined as described previously and classified as: inactive, moderately inactive, moderately active or active [19]. For the analysis on changes in physical activity, we combined both moderately inactive and moderately active into one category so that we had sufficient numbers of participants within each of the changes in physical activity groups. We then defined changes in the condensed CPAI category over two five-year periods (i.e. from baseline to the 5-y follow up and from the 5- to the 10-y follow-up): becoming inactive, staying inactive, staying moderately (in)active, staying active or becoming active.

### Other exposures

Socio-demographic, lifestyle, dietary factors and chronic disease risk factors were determined at each round. Education, based on highest level of education attained, including follow-up, was categorized as: low (intermediate secondary education or less); moderate (intermediate vocational or higher secondary education) or higher (higher vocational education or university) education. Body mass index (BMI) was calculated, from measured weight and height, as weight (in kg) divided by height squared (in metres). Systolic and diastolic blood pressures were also measured and hypertension was defined as systolic blood pressure ≥ 140 mmHg, diastolic blood pressure ≥ 90 mmHg, and/or the use of antihypertensive medication. Diabetes was defined as self-reported diabetes or a random glucose level ≥ 11.1 mmol/l. Hypercholesterolemia was defined as nonfasting total cholesterol ≥6.5 mmol/l and/or self-reported use of cholesterol-lowering medication. A history of cardiovascular disease was defined as present if participants reported myocardial infarction or stroke. Smoking was categorized as never, ex-smoker or current smoker. Alcohol consumption was categorized as non-drinker, light (0–4.9 g/d for both women and men), moderate (5.0–14.9 g/d for women; 5.0–29.9 g/d for men) or heavy drinker (≥15.0 g/d for women; ≥30.0 g/d for men) [20]. Furthermore, diet was assessed by a validated food frequency questionnaire [21, 22]. Participants were asked to report their usual frequency of consumption of 178 food and beverage items over the previous 12 months. Consumption of animal protein (and other nutrient) intakes were calculated by using the Dutch 1996 food-composition database [23].

### Statistical analyses

Baseline characteristics of the study population are described by means, standard deviations, frequencies and percentages. To examine the prospective associations between each of physical activity and 5-year changes in physical activity and eGFR, the technique of generalized estimating equations was adopted because they enable the use of longitudinal linear regression by taking into account correlations within each participant. Both physical activity and 5-year changes in physical activity were included as time-varying covariates.

**Fig 1:**
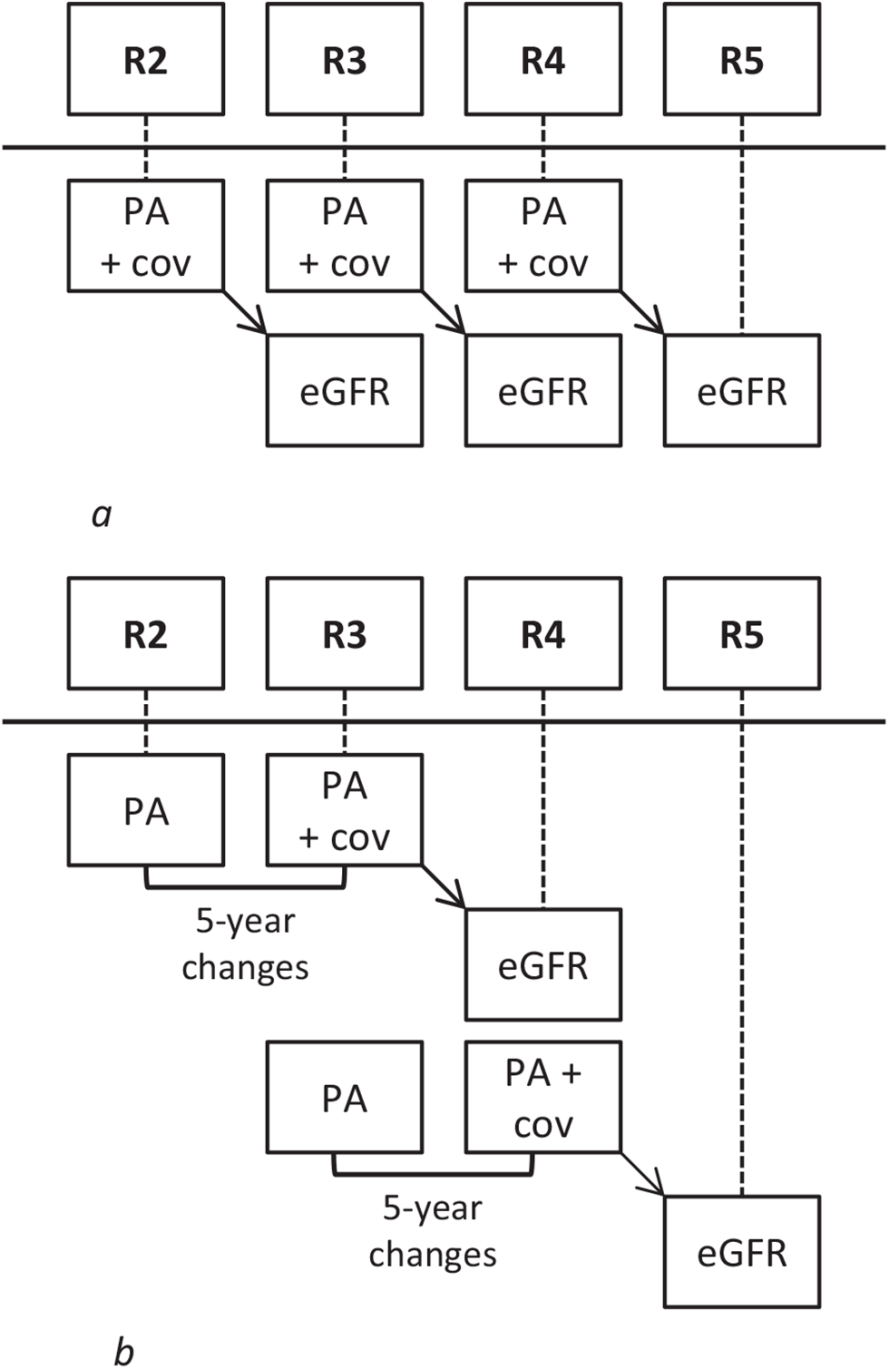
Visualizing the generalized estimating equation regression models with time lag between *a*) physical activity and estimated glomerular filtration rate and *b*) 5-year changes in physical activity and estimated glomerular filtration rate. Abbreviations: R2: round 2, R3: round 3 etc. PA: physical activity, eGFR: estimated glomerular filtration rate. Cov: covariates.

To appropriately deal with issues of reverse causation, time lagged models were used so that both physical activity and 5-year changes in physical activity were associated with kidney function at the subsequent survey (Fig 1). The group of participants reporting being active in the physical activity analysis or becoming active in the 5-year changes in physical activity analysis was set as the reference category. For univariable analyses, models were fitted for (5-year changes in) physical activity and eGFR (Model 1). Multivariable models included age and sex (Model 2), highest attained level of education, BMI, smoking, alcohol consumption, dietary protein from animal sources (Model 3), diabetes, hypertension, hypercholesterolemia and cardiovascular disease (Model 4). Time-varying covariates were used in the physical activity analysis and attained levels of covariates (at R3 and R4, see Fig 1) were used in the 5-year changes in physical activity analysis. Once participants reported diabetes, hypertension or hypercholesterolemia, they were considered to have that condition at all subsequent rounds. In our analyses, collinearity diagnostics between the covariates were found to be low (i.e. all variance inflation factors were around 1.0), indicating an absence of multicollinearity. We also formally tested effect modification by sex and age by adding interaction terms between physical activity and each of sex and age. Finally, we performed a sensitivity analysis, where we repeated the primary analysis, but censored the participants who reported a diagnosis of hypertension, diabetes, hypercholesterolemia or cardiovascular disease from the time of the survey they reported to have the diagnosis. We also repeated analyses using onset of reduced eGFR (<60 mL/min per 1.73 m^2^) or creatinine-based eGFR as the outcomes. The data were analysed using SAS 9.3.

This paper was prepared in accordance with the Strengthening the Reporting of Observational Studies in Epidemiology (STROBE) statement [24].

## Results

**Fig 2:**
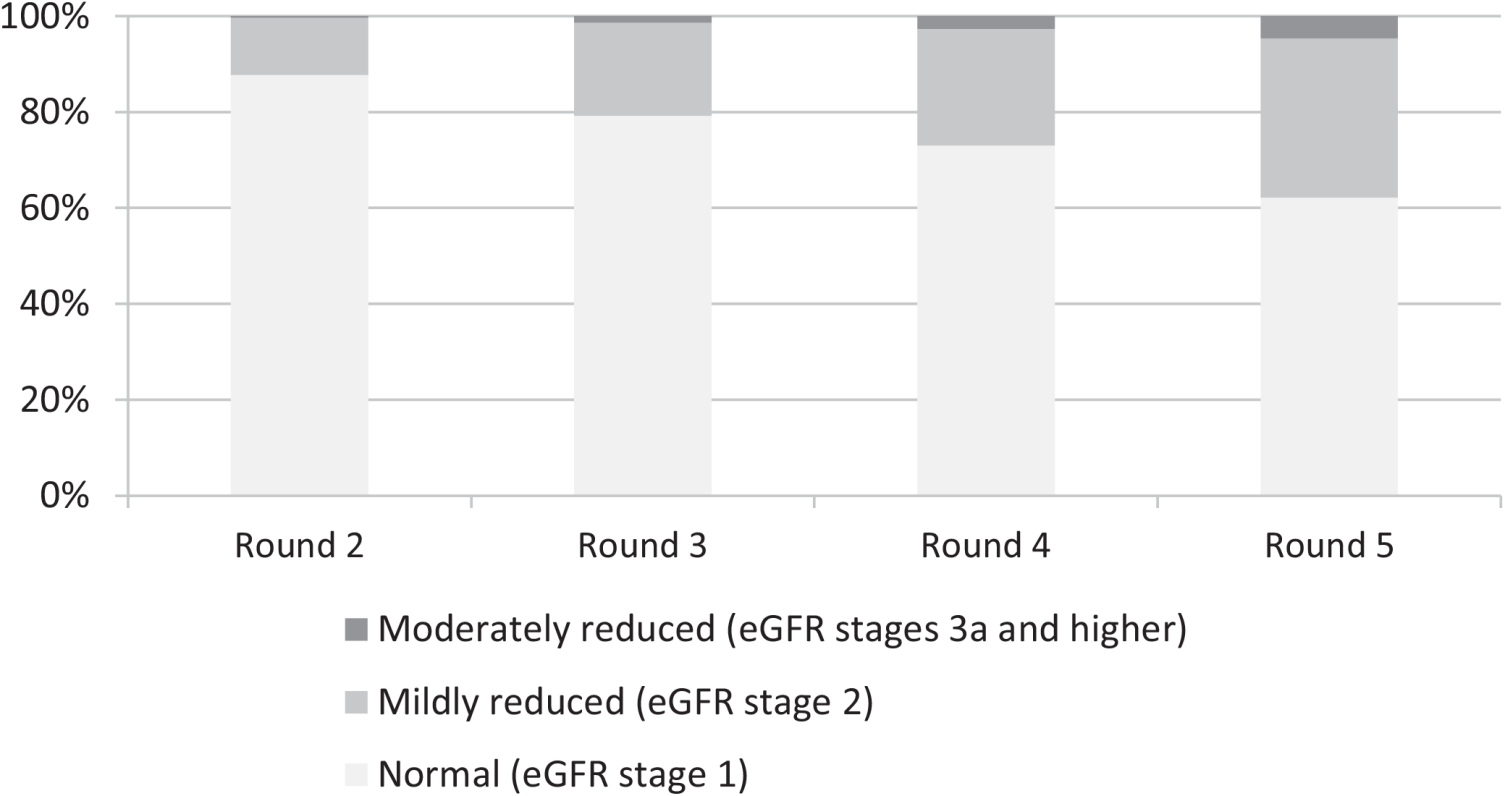
Prevalence of the various eGFR stages during the various screening rounds. Abbreviation: eGFR: estimated glomerular filtration rate.

The mean age of our population was 45.2 (± 9.8) (range 26–65) years, with 47.9% men. Mean baseline eGFR was 107.9 (± 14.5) mL/min per 1.73 m^2^ and 51.9% (N = 2025) of participants were physically active. A further 3.6% (N = 141) were inactive, 18.5% (N = 722) were moderately inactive and 26.0% (N = 1013) were moderately active. Compared with active participants, those who were physically inactive were older, had a higher BMI and were more likely to be men, lower educated, current smoker, non-drinker, and to have diabetes, hypertension, hypercholesterolemia and cardiovascular disease (Table 1). At baseline, 87.6% had a normal (eGFR ≥90 mL/min per 1.73 m^2^), 11.9% had a mildly reduced (eGFR 60–90 mL/min per 1.73 m^2^) and 0.4% had a mild to moderately reduced (eGFR <60 mL/min per 1.73 m^2^) kidney function. The prevalence of a mildly and moderately reduced kidney function increased, so that 15 years after baseline (R5), 62.2% had a normal, 33.2% a mildly reduced and 4.7% a moderately reduced kidney function (Fig 2).

**Table 1:** Characteristics of study population according to physical activity at round 2.

Characteristics	Inactive N = 141 (3.6%)	Moderately inactive N = 722 (18.5%)	Moderately active N = 1013 (26.0%)	Active N = 2025 (51.9%)
	Mean (SD)	Mean (SD)	Mean (SD)	Mean (SD)
Age, y	49.4 (8.6)	46.5 (9.9)	46.1 (9.6)	44.2 (9.6)
BMI, kg/m^2^	26.7 (4.1)	25.7 (4.0)	25.1 (3.6)	25.3 (3.4)
Systolic blood pressure, mm/Hg	127 (18)	126 (17)	125 (16)	125 (16)
Diastolic blood pressure, mm/Hg	82 (12)	80 (11)	80 (11)	80 (11)
Total cholesterol, mmol/L	5.7 (1.0)	5.5 (1.0)	5.5 (1.0)	5.4 (1.0)
Glucose, mmol/L	5.8 (2.3)	5.5 (1.6)	5.2 (1.2)	5.2 (1.2)
Animal protein, g/day	53.4 (16.8)	52.0 (16.4)	52.6 (15.2)	54.9 (16.9)
eGFR, mL/min per 1.73 m^2^	104.2 (15.1)	105.6 (15.2)	107.0 (14.9)	109.5 (14.5)
	N (%)	N (%)	N (%)	N (%)
**Sex**				
Men	85 (60.3)	369 (51.1)	467 (46.1)	963 (47.6)
Women	56 (39.7)	353 (48.9)	546 (53.9)	1062 (52.4)
**Education**				
Low	72 (51.1)	292 (40.4)	413 (40.8)	938 (46.3)
Intermediate	42 (29.8)	210 (29.1)	321 (31.7)	664 (32.8)
High	27 (19.2)	220 (30.5)	279 (27.5)	423 (20.9)
**Smoking**				
Current	59 (41.8)	231 (32.0)	290 (28.6)	586 (28.9)
Past	41 (29.1)	256 (35.5)	390 (38.5)	775 (38.3)
Never	41 (29.1)	235 (32.6)	333 (32.9)	664 (32.8)
**Alcohol consumption**				
Non-drinker	62 (44.0)	280 (38.8)	353 (34.9)	735 (36.3)
Light	11 (7.8)	60 (8.3)	80 (7.9)	207 (10.2)
Moderate	51 (36.2)	294 (40.7)	409 (40.4)	829 (40.9)
Heavy	17 (12.1)	88 (12.2)	171 (16.9)	254 (12.5)
**Diabetes**	8 (5.7)	18 (2.5)	13 (1.3)	18 (0.9)
**Hypertension**	43 (30.5)	194 (26.9)	274 (27.1)	513 (25.4)
**Hypercholesterolemia**	45 (31.9)	180 (25.0)	233 (23.0)	425 (21.0)
**Cardiovascular disease**	6 (4.3)	16 (2.2)	21 (2.1)	28 (1.4)
**Physical activity at round 3**				
Inactive	52 (39.1)	52 (7.6)	31 (3.2)	20 (1.0)
Moderately inactive	45 (33.8)	326 (47.7)	239 (24.6)	174 (9.1)
Moderately active	15 (11.3)	192 (28.1)	363 (37.4)	376 (19.6)
Active	21 (15.8)	114 (16.6)	338 (34.8)	1350 (70.3)
**Physical activity at round 4**				
Inactive	38 (32.8)	39 (6.4)	27 (3.0)	29 (1.6)
Moderately inactive	43 (37.1)	261 (42.9)	194 (21.6)	164 (9.2)
Moderately active	20 (17.2)	186 (30.6)	336 (37.5)	396 (22.3)
Active	15 (12.9)	122 (20.1)	340 (37.9)	1190 (66.9)

### Association between physical activity and kidney function

In the crude model, being less physically active was associated with a lower eGFR. Compared with active participants, the eGFR at the subsequent survey (5 years later) for the other physical activity groups was 0.53 to 2.00 mL/min per 1.73 m^2^ lower (Table 2). However, adjustment for age and sex attenuated the inverse association for physical activity, toward statistically non-significant betas of -0.21 (95% CI -0.65, 0.23), -0.48 (95% CI -1.05, 0.09) and -1.13 (95% CI -2.29, 0.02) mL/min per 1.73 m^2^ in the moderately active, moderately inactive and inactive participants respectively. Additional adjustment for other confounders further attenuated the results. In the fully adjusted model, eGFR at the subsequent survey was 0.22 to 0.57 mL/min per 1.73 m^2^ lower in the less active, as compared to the active participants, all not statistically significant.

**Table 2:** Regression coefficients (mL/min per 1.73 m^2^) and 95% confidence intervals for the association between physical activity and cystatin C-based estimated glomerular filtration rate at the subsequent round, adjusted for time-varying covariates.

	Physical activity			
	Inactive	Moderately inactive	Moderately active	Active
Model 1	-2.00 (-3.18,-0.82)	-0.96 (-1.56,-0.36)	-0.53 (-0.98,-0.08)	Reference
Model 2	-1.13 (-2.29,0.02)	-0.48 (-1.05,0.09)	-0.21 (-0.65,0.23)	Reference
Model 3	-0.57 (-1.70,0.57)	-0.28 (-0.85,0.30)	-0.21 (-0.66,0.23)	Reference
Model 4	-0.57 (-1.70,0.56)	-0.26 (-0.84,0.31)	-0.22 (-0.66,0.22)	Reference

Model 1: crude; Model 2: adjusted for age and sex; Model 3: model 2 and highest attained level of education and time-dependent smoking, alcohol consumption, body mass index and animal protein; Model 4: model 3 and time-dependent diabetes, hypertension, hypercholesterolemia and cardiovascular disease.

### Association between 5-year changes in physical activity and kidney function

The absence of an association between physical activity and eGFR was also observed when we analysed 5-year changes in physical activity (Table 3). After adjustment for age and sex, compared with participants who became active, eGFR at the subsequent survey was a non-significant 0.53 (95% CI -0.26, 1.32) higher and a non-significant 0.01 (95% CI -0.78, 0.76), 2.30 (95% CI -5.64, 1.05) and 0.52 (95% CI -1.25, 0.20) mL/min per 1.73 m^2^ lower in those who stayed active, stayed moderately (in)active, stayed inactive or became inactive respectively. Further adjustment for other confounders did not materially change the results.

**Table 3:** Regression coefficients (mL/min per 1.73 m^2^) and 95% confidence intervals for the associations between 5-year changes in physical activity and cystatin C-based estimated glomerular filtration rate at the subsequent round, adjusted for attained time-varying covariates.

	5-year changes in physical activity				
	Becoming inactive	Staying inactive	Staying moderately (in)active	Staying active	Becoming active
Model 1	-0.40 (-1.14,0.34)	-3.46 (-7.01,0.09)	-0.28 (-1.09,0.53)	1.41 (0.57,2.26)	Reference
Model 2	-0.52 (-1.25,0.20)	-2.30 (-5.64,1.05)	-0.01 (-0.78,0.76)	0.53 (-0.26,1.32)	Reference
Model 3	-0.28 (-1.02,0.45)	-1.22 (-4.63,2.19)	-0.09 (-0.86,0.68)	0.63 (-0.15,1.42)	Reference
Model 4	-0.27 (-1.01,0.47)	-1.10 (-4.50,2.30)	-0.05 (-0.83,0.72)	0.68 (-0.12,1.47)	Reference

Model 1: crude; Model 2: adjusted for age and sex; Model 3: model 2 and highest attained level of education and time-dependent smoking, alcohol consumption, body mass index and animal protein; Model 4: model 3 and time-dependent diabetes, hypertension, hypercholesterolemia and cardiovascular disease. Percentage of people in the various 5-year change groups: 17.05% becoming inactive, 1.54% staying inactive, 31.07% staying moderately (in)active, 35.90% staying active, 14.45% becoming active.

No interaction with sex or age was observed for the association of both physical activity and changes in physical activity with eGFR (P-values ranged from 0.21–0.74). Also, we found similar results in sensitivity analyses where we censored participants who reported diabetes, hypertension, hypercholesterolemia or cardiovascular disease, used onset of reduced eGFR (<60 mL/min per 1.73 m^2^) as the outcome (data not shown) or creatinine-based eGFR (S1 and S2 Tables) as the outcome.

## Discussion

In this prospective population-based cohort study, physical activity levels as well as changes in physical activity over time were not associated with kidney function among adult men and women aged ≥ 20 years.

### Comparisons with other studies

Our findings are not in keeping with the results of most previous population-based studies, which show that physical inactivity is associated with higher risk of reduced eGFR [7–9]. However, their use of the creatinine-based Cockcroft-Gault [8] or the abbreviated Modification of Diet in Renal Disease (MDRD) [7, 9, 25] equations to estimate GFR have been criticized because of their low precision among individuals with normal kidney function [26–28]. Furthermore, all these studies were cross-sectional and the associations observed may therefore reflect reduced physical activity due to presence of chronic kidney failure at baseline, rather than causality. Evidence for this comes from the AusDiab study, a population-based survey that included adults in a comparative age range as our study and found that physical inactivity was positively associated with creatinine-based CKD at baseline, but not with longitudinal outcomes[12]. The latter findings are in agreement with our results of no association between physical activity and kidney function. Apart from the AusDiab study, only two other previous studies examined the prospective association between physical activity and kidney function. In the Cardiovascular Health Study, higher physical activity levels were associated with a 28% lower risk of rapid kidney function decline, defined as loss of >3.0 mL/min/1.73 m^2^ in eGFR per year [10]. However, the mean age of that study population was substantially higher than in our study population (72 versus 46 y at baseline), which may have increased the chance of an already existent limited kidney function. Indeed, whereas the mean eGFR of their study population was 78.4 mL/min per 1.73 m^2^ at baseline, it was 107.9 mL/min per 1.73 m^2^ in our participants at the start of follow-up. Findings from a secondary analysis in our study showed that physical activity was not associated with a rapid kidney function decline either (results not shown). In the NHANES (National Health Examination Study)-II, the relative risk of end-stage kidney disease and CKD-related death was more than two times higher (adjusted RR 2.2, 95% CI 1.3, 3.8) in inactive than in active individuals [11]. However, although this study included adults with a similar age as our study population, the authors investigated physical activity in relation to very severe, or endstage kidney failure, which is different from our study that mostly looked at normal or mildly reduced kidney function. Given the aforementioned considerations it is possible that physical activity can prevent progressive kidney function decline in individuals whose kidney function is already reduced, but not in those with well-preserved kidney function–an interesting avenue for further research.

Another difference with our study is that physical activity in the previous studies included leisure time physical activity only, whereas our measure also included physical activity at work. Additionally, all previous studies measured the exposures at baseline only and any changes during follow-up were therefore not accounted for. This is important because physical activity levels change over time. Effect estimates from the previous studies are therefore likely to be residually confounded.

Our results suggest that 5-year changes in physical activity are not associated with kidney function. However, since this is the first study to date examining the independent effects of changes in physical activity on kidney function, further research is required to see if our findings can be confirmed in other populations. Clearly, it is still important for adults to participate in physical exercise, since regular physical activity has been shown to reduce morbidity [29] and mortality [30] from many other chronic diseases.

### Strengths

Our study has a number of strengths. This was a population-based study, which allows extrapolation of findings to other adult men and women aged 20 years and older. A major contribution of the current analysis was using cystatin C for the primary analysis to estimate kidney function, which has been found to be a better marker of kidney function than creatinine [13]. By measuring all available samples per participant in one assay run, we obtained the most reliable measurement of cystatin C to estimate GFR [17]. Our study also benefits from the use of the CKD-EPI instead of the MDRD formula to estimate kidney function, which is known to provide a more accurate estimate of GFR in individuals with normal or only mildly reduced kidney function [18]. Further strengths lie in the longitudinal nature of the study, with repeated measurements of physical activity and kidney function, long duration of follow-up and the ability to study 5-year changes in physical activity and to adjust for time-varying covariates.

### Limitations

There are also some limitations to our study. First, the assessment of physical activity was based on self-report and some degree of misclassification may exist. However, the questions have been shown to have relatively high reproducibility (weighted kappa statistic = 0.6) and acceptable ranking of participants according to their activity and cardiorespiratory fitness when assessed by objective methods [19]. Furthermore, recent results from our group have shown that this index was clearly associated with both body weight and waist circumference in the Doetinchem Study [31]. It has also been shown to predict all-cause mortality and cardiovascular incidence in the EPIC-Norfolk study [32]. Second, although adjustment for potential confounders had little effect on the estimates, residual confounding due to unmeasured factors, such as conditions that may affect physical activity, such as rheumatoid arthritis and related medication use, may still have been present. Finally, like in other prospective studies, the individuals who were excluded from the analyses, were generally less healthy than those who were included in the analyses (data not shown). Although the differences were small and not expected to change the results substantially, selection bias cannot be excluded.

### Conclusions

In this population-based prospective study of men and women, we did not observe an association between (changes in) physical activity and kidney function. These findings suggest that physical activity modification may not be an important strategy in maintaining kidney health. However, confirmation of our results in different populations of men and women with well-preserved kidney function along with objective measures of physical activity is needed.

## Supporting Information

S1 Table Regression coefficients (mL/min per 1.73 m^2^) and 95% confidence intervals for the association between physical activity and creatinine-based estimated glomerular filtration rate at the subsequent round, adjusted for time-varying covariates. (DOCX)

S2 Table Regression coefficients (mL/min per 1.73 m^2^) and 95% confidence intervals for the associations between 5-year changes in physical activity and creatinine-based estimated glomerular filtration rate at the subsequent round, adjusted for attained time-varying covariates. (DOCX)
