# Supplementary material for: Physical Activity Is not Associated with Estimated Glomerular Filtration Rate among Young and Middle-Aged Adults: Results from the Population-Based Longitudinal Doetinchem Study
Source: PLoS One. 2015 Oct 14;10(10):e0133864. doi: 10.1371/journal.pone.0133864 (PMC4605681; doi:10.1371/journal.pone.0133864)
Supplement: S1 Table — (DOCX) [file pone.0133864.s001.docx]

S1 table Regression coefficients (mL/min per 1.73 m^2^) and 95% confidence intervals for the association between physical activity and creatinine-based estimated glomerular filtration rate at the subsequent round, adjusted for time-varying covariates

|  | **Physical activity** | | | |
| --- | --- | --- | --- | --- |
|  | Inactive | Moderately inactive | Moderately active | Active |
| Model 1 | -1.71 (-2.76, -0.66) | -0.72 (-1.22,-0.23) | -0.46 (-0.86, -0.07) | Reference |
| Model 2 | -0.77 (-1.81,0.27) | -0.25 (-0.73,0.23) | -0.20 (-0.59,0.19) | Reference |
| Model 3 | -0.71 (-1.77,0.34) | -0.20 (-0.68,0.28) | -0.17 (-0.56,0.21) | Reference |
| Model 4 | -0.72 (-1.78,0.33) | -0.18 (-0.67,0.30) | -0.17 (-0.56,0.21) | Reference |

Model 1: crude; Model 2: adjusted for age and sex; Model 3: model 2 and highest attained level of education and time-dependent smoking, alcohol consumption, body mass index and animal protein; Model 4: model 3 and time-dependent diabetes, hypertension, hypercholesterolemia and cardiovascular disease
