# Supplementary material for: Physical Activity Is not Associated with Estimated Glomerular Filtration Rate among Young and Middle-Aged Adults: Results from the Population-Based Longitudinal Doetinchem Study
Source: PLoS One. 2015 Oct 14;10(10):e0133864. doi: 10.1371/journal.pone.0133864 (PMC4605681; doi:10.1371/journal.pone.0133864)
Supplement: S2 Table — (DOCX) [file pone.0133864.s002.docx]

S2 table Regression coefficients (mL/min per 1.73 m^2^) and 95% confidence intervals for the associations between 5-year changes in physical activity and creatinine-based estimated glomerular filtration rate at the subsequent round, adjusted for attained time-varying covariates

|  | **5-year changes in physical activity** | | | | |
| --- | --- | --- | --- | --- | --- |
|  | Becoming inactive | Staying inactive | Staying moderately (in)active | Staying active | Becoming active |
| Model 1 | -0.35 (-1.01,0.30) | -2.72 (-6.37, 0.93) | -0.61 (-1.31,0.09) | 0.65 (-0.07, 1.38) | Reference |
| Model 2 | -0.41 (-1.05,0.23) | -1.50 (-4.95,1.95) | -0.42 (-1.10,0.25) | -0.06 (-0.41,0.62) | Reference |
| Model 3 | -0.38 (-1.02,0.27) | -1.55 (-5.06,1.95) | -0.34 (-1.02,0.34) | -0.08 (-0.76,0.61) | Reference |
| Model 4 | -0.37 (-1.02,0.27) | -1.54 (-5.05,1.97) | -0.33 (-1.01,0.36) | -0.07 (-0.76,0.62) | Reference |

Model 1: crude; Model 2: adjusted for age and sex; Model 3: model 2 and highest attained level of education and time-dependent smoking, alcohol consumption, body mass index and animal protein; Model 4: model 3 and time-dependent diabetes, hypertension, hypercholesterolemia and cardiovascular disease
